# Diabetes Mellitus Duration and Major Adverse Cardiovascular Events Following Percutaneous Coronary Intervention: A Prospective Cohort Study

**DOI:** 10.1155/ijvm/8260099

**Published:** 2026-09-01

**Authors:** Ayesha Tariq, Zohra Bhatti, Amer. H. Khan, Madeeha Laghari, Bandeh Ali Talpur, Muhammad Aamir, Khalid Hussain

**Affiliations:** ^1^ Department of Pharmacy, University of Lahore, Lahore, Pakistan, uol.edu.pk; ^2^ Department of Pharmacy Practice, Kulliyyah of Pharmacy, International Islamic University Malaysia, Kuantan, Malaysia, iium.edu.my; ^3^ Department of Pharmacy Practice, Faculty of Pharmacy, University of Tabuk, Tabuk, Saudi Arabia, ut.edu.sa; ^4^ Department of Clinical Pharmacy, School of Pharmaceutical Sciences, University Sains Malaysia, Penang, Malaysia, usm.my; ^5^ School of Computer Science and Statistics, Trinity College Dublin, Dublin, Ireland, tcd.ie

**Keywords:** duration of diabetes mellitus, major adverse cardiovascular events, percutaneous coronary intervention, Type 2 diabetes mellitus

## Abstract

**Objective:**

This study is aimed at evaluating the impact of Type 2 diabetes mellitus (T2DM) duration on major adverse cardiovascular events (MACEs) after percutaneous coronary intervention (PCI).

**Methods:**

A single‐center prospective cohort study was conducted in which 465 T2DM patients undergoing PCI and completing the 1‐year follow‐up were enrolled. Patients were divided into three groups based on duration of DM: < 5 years, 5–10 years, and > 10 years. The primary endpoint was MACE, a composite of nonfatal myocardial infarction (MI), stroke, coronary artery bypass grafting (CABG), repeat PCI, and mortality.

**Results:**

The incidence of MACE increased progressively with longer diabetes duration: 3.7% (< 5 years), 8.1% (5–10 years), and 13.9% (> 10 years), with particularly higher rates of stroke (0.6%–3.0%) and repeat PCI (1.8%–6.0%) among T2DM patients after PCI. The Kaplan–Meier analysis demonstrated a clear stepwise rise in cumulative MACE incidence over 12 months, with the highest event rates observed in patients with diabetes for more than 10 years (log‐rank *p* < 0.004). Consistently, Cox proportional hazards models showed significantly elevated risk compared with the < 5‐year group, with adjusted hazard ratios of 3.16 (95% CI: 1.06–9.47, *p* = 0.039) for 5–10 years and 4.37 (95% CI: 1.59–12.01, *p* = 0.004) for > 10 years.

**Conclusion:**

Patients with longer duration (> 10 years) of T2DM had the highest incidence and hazard of adverse cardiovascular events compared to shorter duration (< 5 years and 5–10 years). These results suggest that targeted preventive strategies and closer post‐PCI monitoring are essential for patients with prolonged diabetes duration.

## 1. Introduction

Cardiovascular diseases (CVDs) are the main cause of poor life expectancy and increased mortality rate in people with diabetes mellitus (DM) [1]. DM patients have a higher incidence of coronary heart disease (CHD), myocardial infarction (MI), hypertension, and stroke compared to individuals without diabetes [2]. Globally, the prevalence of CVD in diabetic patients is 34.8%, with 16% dying from stroke and 68% of cardiac complications [3, 4]. In Pakistan, the prevalence of diabetes is estimated to be around 26.7% among adults, one of the highest in the world, and cardiovascular complications remain the leading cause of mortality among these patients. This illustrates how the global burden of CVD in diabetes is particularly magnified in Pakistan [5, 6].

This increased risk can be attributed to several risk factors, including obesity, metabolic syndrome, chronic inflammation, and oxidative stress [7], which accelerate cardiovascular damage in prolonged diabetes and contribute to adverse events such as MI and stroke [8]. The American Diabetes Association (ADA) demonstrated that people with diabetes were not appropriately and effectively guided regarding the increasing burden of CVD, which is important to improve the clinical outcomes and help them prevent the incidence of major adverse cardiovascular events (MACEs) [9]. Although strong evidence links diabetes to cardiovascular risk, glycemic control management and secondary prevention vary across populations. Global guidelines recommend multifactorial control of glucose, lipids, and blood pressure to reduce CVD burden [10, 11]. Yet implementation remains limited in low‐ and middle‐income countries like Pakistan due to poor access, delayed diagnosis, and suboptimal adherence.

The duration of diabetes in individuals with coronary artery disease (CAD) is also related to clinical outcomes since DM is a long‐term, progressive illness that causes microvascular or macrovascular damage [12]. Previous research found that a longer duration of Type 2 diabetes mellitus (T2DM) was significantly correlated with cardiovascular events and raised the probability of dying from CAD. Thus, the presence of T2DM for a long duration (> 10.5 years) is strongly linked to severe cardiovascular outcomes even after percutaneous coronary intervention (PCI) [13]. Studies suggest that, alongside hyperglycemia and impaired repair mechanisms of the body, patients with a longer history of diabetes often exhibit more severe coronary lesions and poorer collateral circulation, which can compromise the ability of the heart to compensate for impaired blood flow during ischemic events [14, 15]. In South Asian populations, including Pakistan, earlier onset and longer duration of T2DM have been reported compared to Western countries, which may predispose patients to more aggressive CAD and worse PCI outcomes [16].

Published data have highlighted that the incidence of MACE increases with the duration of DM, and a strong relation was found between T2DM and adverse outcomes after PCI [17]. Studies have shown that, controlling for demographics and other comorbidities, the risk of CHD increases by 1.38 times for each 10‐year increase in the duration [18]. A comprehensive meta‐analysis supported these findings by analyzing results from multiple trials and concluded that the frequency of stent thrombosis and restenosis was higher among T2DM patients after PCI [17]. However, most available data originate from Western or East Asian populations, leaving South Asian cohorts underrepresented despite a disproportionately high diabetes burden. The lack of regional evidence limits context‐specific risk assessment and the development of preventive strategies.

While examining the effects of diabetes on CAD and revascularization operations [19], it is important to consider the duration of diabetes to assess the target population for the secondary prevention [14, 20]. In Pakistan, the majority of patients present late with poorly controlled diabetes and advanced CAD, which may further increase their risk of MACE after PCI [21]. However, prospective data evaluating the relationship between diabetes duration and PCI outcomes in this region are limited. Addressing this gap is essential for improving cardiovascular outcomes through locally relevant evidence to guide prevention strategies, patient counseling, and post‐PCI diabetes management in resource‐limited settings. Therefore, the present study addresses an important research gap by evaluating the association between T2DM duration and the incidence of MACE following PCI and providing locally relevant evidence to guide risk stratification and secondary prevention in Pakistani patients.

## 2. Methodology

### 2.1. Study Design, Setting, and Duration

A prospective cohort study design was adopted to conduct the study. A key advantage of this design is that it tracks a cohort of individuals over time to gather information on exposures and monitor outcomes. The present study was performed at the Punjab Institute of Cardiology, Lahore, a tertiary health care institute of Punjab Province, Pakistan. Data were collected from patients admitted to hospital wards for PCI from September 20, 2023, to January 20, 2024, and follow‐up was conducted for the next 12 months of each patient from the date of enrollment.

Participants of any gender aged ≥ 18 years were included, with acute MI (STEMI/NSTEMI and unstable angina) and postprocedural TIMI‐3 flow, with T2DM. Participants were excluded if they had a prior history of MI, CABG, or PCI; hemoglobinopathies; hemolytic anemia; HIV infection; chronic kidney disease; pericardial disease; valvular heart disease; cardiogenic shock; ST‐elevation ECG without clear signs of CAD, such as acute myocarditis, early repolarization, or Takotsubo cardiomyopathy; or T1DM.

### 2.2. Study Sample

**Figure 1:**
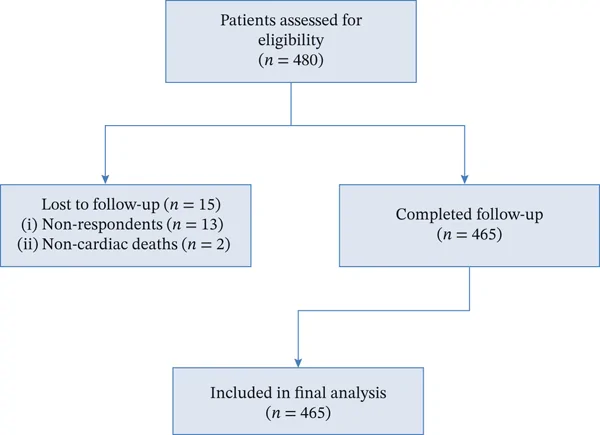
CONSORT‐style flow diagram showing patient enrollment, follow‐up, and final sample for analysis.

A standard cohort study formula using Epi‐Info was used to determine the required sample size. The researchers reported that a sample size of 480 was appropriate, based on the prevalence of MACE in T2DM patients undergoing PCI, with a 95% confidence interval (CI) and 80% power. A total of 480 patients were initially enrolled; however, 15 patients were lost during follow‐up, resulting in a final analytical sample of 465 participants. A CONSORT‐style flow diagram summarizing participant enrollment, follow‐up, and final inclusion is presented in Figure 1. A consecutive sampling technique was adopted to choose sample units, in which all eligible participants were enrolled during the study period until the required sample size was achieved to represent the whole population across Punjab Province, Pakistan.

### 2.3. Study Instrument

The data collection tool was developed through a multiphase process to ensure validity and appropriateness for assessing clinical and demographic characteristics of T2DM patients undergoing PCI with drug‐eluting stent (DES) and also to evaluate MACE during follow‐up.

#### 2.3.1. Literature Review and Conceptual Framework

A thorough literature review of previous studies was conducted and followed clinical guidelines to identify key variables related to patients′ sociodemographic factors, previous medical records, factors that accelerate CAD, glycemic control (hemoglobin A1c [HbA1c]) monitoring, and cardiovascular outcomes. This guided the main structure and scope of the data collection form to ensure that it included all the required domains to fulfill the study criteria.

#### 2.3.2. Initial Drafting

A structured data collection form was developed in consultation with a cardiology expert and with the guidance of academic supervisors. It comprised eight sections:

• Sociodemographic data (age, gender, BMI, education, income, etc.)
• Clinical characteristics (angiographic findings, stent details)
• Medical history (e.g., diabetes duration, hypertension, dyslipidemia, smoking status, and family history)
• Risk factors for CAD (smoking, hypertension, dyslipidemia, etc.)
• Laboratory investigations (HB, RBG, HbA1c, etc.)
• Pharmacotherapy after PCI
• Outcomes during follow‐up (nonfatal MI, repeat PCI, CABG, stroke, and cardiac death)

#### 2.3.3. Translation and Expert Validation

The structured data collection form included data extracted from patient profiles, which required no translation, as well as patient‐reported outcomes, which were administered in Urdu and back‐translated to ensure linguistic and cultural accuracy. Face and content validity were assessed by a panel of five experts (two cardiologists, one epidemiologist, and two senior faculty members) who reviewed the form for clarity, relevance, and comprehensibility. Minor revisions were made to improve wording, formatting, medical terminology, and the logical flow of questions, ensuring the tool was clear and appropriate for participants with varying literacy levels.

#### 2.3.4. Pilot Testing

To evaluate the flow, clarity, and feasibility of the prepared data collection form in a real clinical setting, a pilot test was performed with 35 eligible participants, who were not included in the main sample of 480. Their feedback was recorded regarding questions that were difficult to understand or lacked clarity, and all the important modifications were made in the final study tool.

#### 2.3.5. Final Refinement and Approval

A final version of the data collection form was approved by the supervisory committee after incorporating all relevant suggestions from experts and participants, and it was used for data collection.

### 2.4. Data Collection Procedure

A systematic and standardized process was adopted to maintain the integrity, consistency, and reliability of data collection.

#### 2.4.1. Collection of Baseline Information

At baseline, essential information of each participant, including demographic, clinical, and laboratory data, was collected using the structured questionnaire to provide a foundation for outcome assessment.

#### 2.4.2. Patient Stratification and Grouping

Data were collected from participants suffering from T2DM, taking oral hypoglycemic medications, or insulin, or those having a history of diabetes. According to the ADA diagnostic criteria for diabetes and clinical presentation, we shall consider diabetic people with HbA1c equal to or more than 6.5% to be diabetic. The duration of diabetes was calculated from the time of diagnosis to PCI, and patients were stratified into three groups: < 5 years, 5–10 years, and > 10 years. These group definitions were applied consistently throughout the study.

#### 2.4.3. Questionnaire Administration

Key patient information, including patient demographics, medical history, and risk factors, was extracted from patient profiles and recorded on a structured data collection form. Clinical outcomes were obtained directly from patients using the same form. Standardized instructions ensured consistency, and for patients unable to attend in person during follow‐up, outcomes were collected via telephone to maximize completeness.

#### 2.4.4. Ensuring Data Accuracy

Collected data were systematically entered and verified to ensure accuracy. Data entry and verification procedures, including cross‐checking by independent researchers, ensured data integrity. Research team members involved in verification were trained in data validation and handling incomplete responses.

#### 2.4.5. Primary Outcomes of the Study

Clinical outcomes, including nonfatal MI, stroke, CABG, repeat PCI, and cardiac death, were systematically recorded after 6 months and again after 12 months postprocedure to ensure uniform follow‐up.

#### 2.4.6. Handling of Missing Data

During follow‐up, 15 patients were lost, including 13 nonrespondents and 2 who had noncardiac death. A complete case analysis approach was used, and these patients were excluded from the final outcome analysis. No imputation was performed, as the proportion of missing data was minimal and unlikely to significantly influence the results.

### 2.5. Statistical Analysis

Data were analyzed using Statistical Package for Social Sciences (IBM SPSS Statistics for Windows, Version 21.0, Armonk, New York: IBM Corp.). A significance threshold of *p* < 0.05 was applied to determine statistical significance in all tests. To ensure the reliability and accuracy of results, the data were double‐checked for outliers and errors. Descriptive statistics were used to summarize data. Continuous variables were expressed as mean ± SD, and categorical variables were presented as absolute frequencies and percentages. Inferential statistics based on the normality of the data were applied for analysis to evaluate the association of duration of T2DM with MACE. Comparisons were performed across groups of DM duration, along with demographic and baseline clinical characteristics of responding participants using the chi‐square tests for categorical variables and the Kruskal–Wallis test for continuous variables. Furthermore, survival analysis was performed using the unadjusted Kaplan–Meier analysis (outcomes reported as log‐rank test) and Cox proportional hazard analysis, both as univariate (unadjusted) and multivariate (adjusted) analysis, expressed as adjusted hazard ratios (HRs) with 95% CIs to estimate time‐to‐event data and understand the particular risk of an event (MACE) during a 12‐month follow‐up. The proportional hazards assumption was assessed by including time‐dependent covariates in the Cox regression model.

## 3. Results

### 3.1. Sociodemographic and Baseline Clinical Characteristics of Patients Stratified by Duration of T2DM

A total of 480 patients were assessed for eligibility. During follow‐up, 15 patients were lost (13 nonrespondents and 2 noncardiac deaths), resulting in a final sample of 465 participants (Figure 1). The sociodemographic and baseline clinical characteristics of all 465 participants included in this study are presented in Tables 1, 2, 3, and 4 and were stratified by duration of T2DM: < 5 years, 5–10 years, and > 10 years among T2DM patients following PCI. Male participants were predominant in all groups. Age showed a significant association (*p* < 0.001), with most participants aged ≥ 45 years. BMI was borderline significant (*p* = 0.056); most patients in the 5–10‐year group had normal BMI (< 25). Education and monthly income were not significantly associated, though secondary education and income ≤ 60,000 PKR were most common (Table 1).

**Table 1:** Sociodemographic characteristics of patients stratified by duration of T2DM.

Sociodemographic characteristics		< 5 years (*n* = 163)	5–10 years (*n* = 136)	> 10 years (*n* = 166)	Total (*n* = 465)	*p* value
Gender	Male	116 (71.2)	100 (73.5)	114 (68.7)	330 (71.0)	0.651
	Female	47 (28.8)	36 (26.5)	52 (31.3)	135 (29.0)	

Age	≥ 45 years	129 (79.2)	124 (91.2)	156 (94.0)	409 (88.0)	< 0.001
	< 45 years	34 (20.8)	12 (8.8)	10 (6.0)	56 (12.0)	

BMI	< 25 kg/m^2^ (normal)	87 (53.3)	90 (66.2)	89 (53.6)	266 (57.2)	0.056
	≥ 25 kg/m^2^ (overweight/obese)	76 (46.6)	46 (33.8)	77 (46.4)	199 (42.8)	

Education	≤ Primary	59 (36.2)	57 (41.9)	74 (44.6)	190 (40.9)	0.562
	≥ Secondary	104 (63.8)	79 (58.1)	92 (55.4)	275 (59.1)	

Monthly income	≤ 60,000 (low/medium)	119 (73.0)	99 (72.8)	121 (72.9)	339 (72.9)	0.973
	> 60,000 (high)	44 (27.0)	37 (27.2)	45 (27.1)	126 (27.1)	

*Note:* Values are presented as *n* (%), with percentages calculated within each diabetes duration group. Comparisons between groups were performed using the chi‐square test.

Abbreviation: BMI, body mass index.

**Table 2:** Baseline clinical characteristics of patients stratified by duration of T2DM.

Baseline clinical characteristics		< 5 years (*n* = 163)	5–10 years (*n* = 136)	> 10 years (*n* = 166)	Total (*n* = 465)	*p* value
Ejection fraction (%)		50.76 ± 10.40	53.10 ± 10.03	50.86 ± 9.25	51.48 ± 9.93	0.064
Hemoglobin (g/dL)		13.30 ± 1.34	13.36 ± 1.31	13.31 ± 1.22	13.32 ± 1.29	0.872
Random blood glucose level (mg/dL)		268.08 ± 77.95	271.80 ± 75.80	292.83 ± 84.6	278 ± 80.18	0.014

HbA1c at the time of PCI	≤ 7.0%	54 (33.1)	43 (31.6)	45 (27.1)	142 (30.5)	0.218
	7.1%–8.0%	85 (52.1)	69 (50.7)	83 (50.0)	237 (51.0)	
	> 8.0%	24 (14.7)	24 (17.6)	38 (22.9)	86 (18.5)	

ECG	Unstable angina	17 (10.4)	11(8.1)	20 (12.0)	48 (10.3)	0.052
	NSTEMI	93 (57.1)	89 (65.4)	95 (57.2)	277 (59.6)	
	STEMI	53 (32.5)	36 (26.5)	51 (30.7)	140 (30.1)	

Therapy of DM	ITDM	38 (23.3)	22 (16.2)	46 (27.7)	106 (22.8)	0.047
	NITDM	125 (76.7)	114 (83.8)	120 (72.3)	359 (77.2)	

Vessels showing CAD	Single vessel	73 (44.8)	32 (23.5)	43 (25.9)	148 (31.8)	< 0.001
	Multivessel (DVD + TVD)	90 (55.2)	104 (76.5)	123 (74.1)	317 (68.2)	

Number of stents passed	One	73 (44.8)	38 (27.9)	38 (22.9)	149 (32.0)	< 0.001
	≥ Two	90 (55.2)	98 (72.1)	128 (77.1)	316 (68.0)	

Type of procedure	Emergency	39 (23.9)	19 (14.0)	25 (15.1)	83 (17.8)	0.041
	Elective	124 (76.1)	117 (86.0)	141 (84.9)	382 (82.2)	

*Note:* Values are presented as mean ± standard deviation for continuous variables and *n* (%) for categorical variables. Percentages are calculated within each diabetes duration group. Comparisons between groups were performed using the chi‐square test for categorical variables and the Kruskal–Wallis test for continuous variables.

Abbreviations: CAD, coronary artery disease; DM, diabetes mellitus; DVD, double‐vessel disease; ECG, electrocardiogram; ITDM, insulin‐treated diabetes mellitus; NITDM, noninsulin‐treated diabetes mellitus; NSTEMI, non–ST elevation myocardial infarction; STEMI, ST elevation myocardial infarction; TVD, triple‐vessel disease.

**Table 3:** Baseline medical history and risk factors of patients stratified by duration of T2DM.

Medical history/risk factors		< 5 years (*n* = 163)	5–10 years (*n* = 136)	> 10 years (*n* = 166)	Total (*n* = 465)	*p* value
Medical history						
Cardiovascular diseases	Yes	77 (47.2)	75 (55.1)	88 (53.0)	240 (51.6)	0.357
Heart failure	Yes	47 (28.8)	30 (22.1)	44 (26.5)	121 (26.0)	0.407
Cerebrovascular disease	Yes	8 (4.9)	7 (5.1)	12 (7.2)	27 (5.8)	0.618
COPD	Yes	14 (8.6)	10 (7.4)	16 (9.6)	40 (8.6)	0.780
Peripheral artery disease	Yes	16 (9.8)	16 (11.8)	20 (12.0)	52 (11.2)	0.787
Diabetic ulcer	Yes	8 (4.9)	8 (5.9)	10 (6.0)	26 (5.6)	0.894
Diabetic neuropathy	Yes	39 (23.9)	27 (19.9)	34 (20.5)	100 (21.5)	0.641
COVID‐19	Yes	20 (12.3)	24 (17.6)	24 (14.5)	68 (14.6)	0.423
COVID‐19 vaccination	Yes	68 (41.7)	51 (37.5)	66 (39.8)	185 (39.8)	0.795
Risk factors						
Smoker	Yes	58 (35.6)	44 (32.4)	70 (42.2)	172 (37)	0.192
Hypertension	Yes	84 (51.5)	85 (62.5)	82 (49.4)	251 (54)	**0.040**
Dyslipidemia	Yes	36 (22.1)	22 (16.2)	40 (24.1)	98 (21.1)	0.226
Family history of CAD	Yes	54 (33.1)	26 (19.1)	42 (25.3)	122 (26.2)	**0.022**
Alcohol intake	Yes	8 (4.9)	7 (5.1)	7 (4.2)	22 (4.7)	0.923

*Note:* Values are presented as *n* (%), with percentages calculated within each diabetes duration group. Comparisons between groups were performed using the chi‐square test.

Abbreviations: DM, diabetes mellitus; CAD, coronary artery disease; COPD, chronic obstructive pulmonary disease; COVID‐19, coronavirus disease 2019; CVD, cardiovascular disease.

**Table 4:** Baseline medications of patients stratified by duration of T2DM.

Medications		<5 years (n = 163)	5–10 years (n = 136)	>10 years (n = 166)	Total (n = 465)	p‐value
Oral Hypoglycemic Drugs	Biguanides (Metformin)	33 (20.2)	17 (12.5)	46 (27.7)	96 (20.6)	0.062
	SGLT‐2 Inhibitors	2 (1.2)	3 (2.2)	5 (3.0)	10(2.2)	
	Sulfonylureas & DPP‐4 Inhibitors	6 (3.7)	7 (5.1)	4 (2.4)	17 (3.7)	
	Combination Therapy	108 (66.2)	100 (73.5)	104 (62.7)	312 (67.1)	
Lipid Lowering Drugs	Atorvastatin	35 (21.5)	30 (22.1)	31 (18.7)	96 (20.6)	0.579
	Rosuvastatin	126 (77.3)	106 (77.9)	132 (79.5)	364 (78.3)	
Rate Limiting Drugs	Beta Blockers	146 (89.6)	114 (83.8)	149 (89.8)	409 (88.0)	0.128
	None or Other	17 (10.4)	22 (16.2)	17 (10.2)	56 (12.0)	
Antihypertensive Drugs	CCB & CCB+ ACI	48 (29.4)	51 (37.5)	64 (38.6)	163 (35.1)	**0.008**
	ACI, ARB & Other	36 (22.1)	36 (26.5)	17 (10.2)	89 (19.1)	
Antianginal Drugs	Nitrates	54 (33.1)	48 (35.3)	60 (36.1)	162 (34.8)	0.324
	Nitrates + Ranolazine or Trimetazidine	35 (21.5)	20 (14.7)	40 (24.1)	95 (20.8)	
Diuretics	Furosemide	25 (15.3)	18 (13.2)	27 (16.3)	70 (15.1)	0.772
	Furosemide + Aldactone	29 (17.8)	19 (14.0)	29 (17.5)	77 (16.6)	
Antiplatelets	Disprin + Clopidogrel	158 (96.9)	135 (99.3)	163 (98.2)	456 (98.1)	0.342
	Disprin + Ticagrelor	5 (3.1)	1 (0.7)	3 (1.8)	9 (1.9)	

*Note:* Values are presented as *n* (%), with percentages calculated within each diabetes duration group. Comparisons between groups were performed using the chi‐square test.

Abbreviations: ACE, angiotensin‐converting enzyme; ARB, angiotensin receptor blocker; CCB, calcium channel blocker; DM, diabetes mellitus; DPP‐4, Dipeptidyl Peptidase‐4; SGLT‐2, Sodium–Glucose Cotransporter‐2.

The mean ejection fraction was highest in the 5–10‐year group and lowest in the < 5‐year group. RBG was significantly associated with DM duration (*p* = 0.014), highest in > 10 years; HbA1c > 8*%* also increased with duration, but not significantly. ECG showed borderline significance (*p* = 0.052), with NSTEMI common among respondents. Insulin therapy was more prevalent in > 10 years. Multivessel disease and ≥ 2 stents were more common in longer durations (*p* < 0.001) (Table 2).

No significant differences were found in medical history variables (*p* > 0.05) stratified by groups of T2DM, but among risk factors, hypertension and family history of CAD showed significance (*p* = 0.040 and *p* = 0.022) (Table 3).

Among medications, only antihypertensives differed significantly (*p* = 0.008), with the CCB + ACI combination being common in individuals aged 5–10 years. Other medications showed similar use across groups (*p* > 0.05) (Table 4).

### 3.2. Impact of T2DM Duration on MACE Following PCI

**Figure 2:**
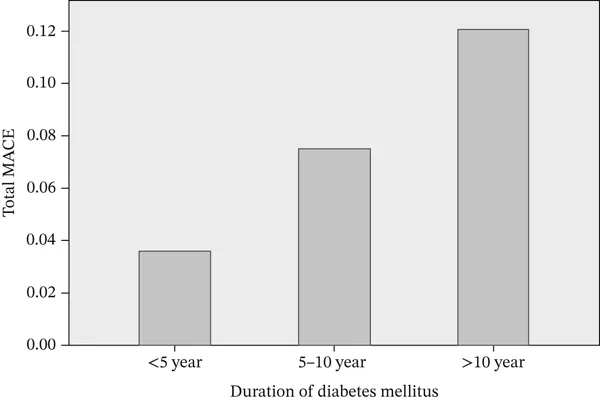
Impact of duration of T2DM groups (< 5 years, 5–10 years, and > 10 years) on incidence of total MACE following PCI.

Results reported that the duration of T2DM and MACE events was directly associated. Patients with shorter duration (< 5 years) reported relatively fewer events, resulting in a total MACE rate of 3.7%. Patients with a DM duration of 5–10 years had more events, with nonfatal MI, CABG, repeat PCI, stroke, and death contributing to a total rate of 8.1%. Those with longer duration (> 10 years) reported the highest events, with nonfatal MI, CABG, repeat PCI, stroke, and deaths leading to the highest total MACE rate of 13.9% (Table 5). These results are graphically presented in Figure 2.

**Table 5:** Impact of T2DM duration on MACE following PCI.

Major adverse cardiovascular events	Duration of DM		
	< 5 years (*n* = 163)	5–10 years (*n* = 136)	> 10 years (*n* = 166)
Nonfatal MI	1 (0.6)	2 (1.5)	2 (1.2)
CABG	1 (0.6)	2 (1.5)	3 (1.8)
Repeat PCI	3 (1.8)	1 (0.7)	10 (6.0)
Stroke	1 (0.6)	3 (2.2)	5 (3.0)
Death	0 (0.0)	3 (2.2)	3 (1.8)
Total MACE	6 (3.7)	11 (8.1)	23 (13.9)

*Note:* Values are presented as *n* (%). Percentages are calculated within each group.

Abbreviations: CABG, coronary artery bypass grafting; MACE, major adverse cardiovascular event; MI, myocardial infarction; PCI, percutaneous coronary intervention.

### 3.3. Association of T2DM Duration With Incidence of MACE Following PCI

**Figure 3:**
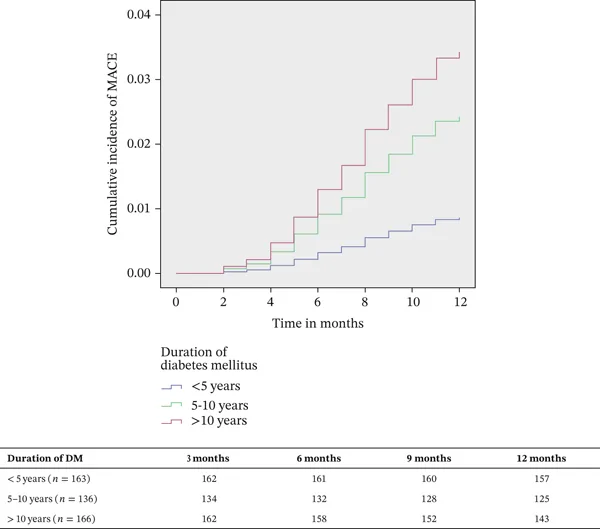
The Kaplan–Meier curves showing the cumulative incidence of major adverse cardiovascular events (MACEs) stratified by duration of T2DM. Differences between groups were assessed using the log‐rank test. Values represent the number of patients at risk at each follow‐up interval. The cumulative incidence of MACE was 3.7% (6/163) in patients with T2DM duration < 5 years, 8.1% (11/136) in those with 5–10 years, and 13.9% (23/166) in those with > 10 years.

Survival analysis was performed to estimate time‐to‐event data and understand the particular risk of an event (MACE) during a 12‐month follow‐up stratified by duration of T2DM after PCI, using the Kaplan–Meier survival curve and Cox proportional hazard analysis (Figure 3 and Table 6).

**Table 6:** Association of T2DM duration with incidence of MACE following PCI.

Duration of DM	*n*	Events (MACE)	Unadjusted			Adjusted		
			HR	95% CI	*p*value	HR	95% CI	*p* value
< 5 years (ref.)	163	6	1.000	—	—	1.000	—	—
5–10 years	136	11	2.217	0.820–5.995	0.117	3.164	1.058–9.465	0.039
> 10 years	166	23	3.940	1.604–9.677	0.003	4.374	1.594–12.005	0.004

*Note:* Reference category: < 5 years. Adjusted for age, gender, BMI, smoking status, hypertension, dyslipidemia, history of cardiovascular disease, HbA1c level, left ventricular ejection fraction, diabetes therapy, oral hypoglycemic agents, and antiplatelet therapy. The relatively wide confidence intervals reflect the limited number of events and should be interpreted with caution.

Abbreviations: CI, confidence interval; HR, hazard ratio; MACE, major adverse cardiovascular event.

The Kaplan–Meier analysis illustrated that patients with > 10 years of T2DM had the highest cumulative incidence of MACE, followed by 5–10 years, while the lowest incidence was reported among patients with < 5 years of T2DM. The log‐rank test value (*p* = 0.004) confirmed that the difference among categories of T2DM duration groups was statistically significant. Cox proportional hazard analysis further supported these findings. Compared to < 5 years (HR = 1), patients with 5–10 years reported an increased HR that became significant after adjustment (*p* = 0.039), while those with > 10 years reported statistically significant and higher HRs in both unadjusted (*p* = 0.003) and adjusted (*p* = 0.004) models. The proportional hazards assumption was assessed using time‐dependent covariates, and no significant violation was observed, indicating that the assumption was satisfied and the HRs remained constant over the follow‐up period.

## 4. Discussion

The findings of this study indicate a linear relationship between T2DM duration and the risk of major MACE, with event rates approximately doubling every 5 years, highlighting that patients with a longer duration of T2DM were more prone to cardiovascular events over time; this aligns with previous evidence showing prolonged diabetic disease as a strong predictor of cardiovascular complications [22, 23]. This association was evaluated and confirmed by utilizing a comprehensive statistical methodology. The use of the chi‐square and Kruskal–Wallis tests for preliminary associations, followed by survival analysis, establishes a strong analytical framework that adheres to the most recent best practices in cardiovascular outcomes research. This multimodal approach has also been applied in recent studies to investigate the temporal links between cardiovascular outcomes and metabolic variables [24].

This study analyzed the characteristics of diabetic patients undergoing PCI by stratifying them based on their duration of diabetes. A statistically significant association was found between age and duration of diabetes (*p* < 0.001), with a greater proportion of patients aged over 60 years in the group with a duration of diabetes of more than 10 years. These results are consistent with previous studies [25], indicating a higher risk of complications in older individuals with prolonged diabetes. TVD and the number of stents used were also found to be significantly associated with the duration of diabetes (*p* < 0.05), with both being more common in the > 10‐year T2DM group. These findings are in agreement with studies [18, 26–28], which reported that longer duration of diabetes is associated with more severe CAD and a greater need for multiple stents. Regarding medication use, a statistically significant association was observed between insulin use and duration of diabetes (*p* < 0.001), with a higher prevalence of insulin therapy in patients with a diabetes duration of > 10 years. This is consistent with prior findings [29], which explained the progressive nature of *β*‐cell dysfunction in long‐standing diabetes necessitating insulin therapy [30–32]. The ORIGIN trial also indicated a possible link between insulin use and adverse cardiovascular outcomes in high‐risk patients [33]. In terms of antihypertensive regimens, a statistically significant association was found between the use of specific antihypertensive combinations and duration of diabetes (*p* = 0.035), with ACEI + CCB being more commonly used in patients with a longer duration of diabetes. These findings are supported by existing guidelines and literature [34, 35], which recommend these combinations for diabetic hypertensive patients. These results are consistent with the findings of large trials [36, 37], which demonstrated that patients with long‐standing diabetes have more complex disease, require more intensive interventions, and may have less favorable outcomes.

The findings of this research indicate a clear graded relationship between the duration of diabetes and MACE, which increases from 3.7% in patients with diabetes lasting less than 5 years to 13.9% in those exceeding 10 years. These results reinforce and expand upon previous studies regarding the link between diabetes duration and cardiovascular risk [38]. The high prevalence of repeat PCI (6.0%) in our > 10‐year diabetic group supports findings associated with accelerated atherosclerosis and exceeded the 4.2% observed in the TWILIGHT‐DM substudy, indicating more aggressive restenosis [39]. Mortality rates (0%, 2.2%, and 1.8%) were slightly lower than previously reported [40], and stroke rates (0.6%–3.0%) suggest cumulative microvascular damage [41]. Consistent with earlier trials, patients with DM > 10 years had a 42% greater risk of MACE [42], with every 5‐year increase linked to an 18% rise in cardiovascular death and 15% in HF hospitalization [43]. A 2.1‐fold increased risk of stent thrombosis was also reported post‐PCI [44].

Furthermore, this association was evident in relation to time‐to‐event outcomes evaluated by survival analysis. Our Kaplan–Meier results (log‐rank *p* < 0.004) showed clear duration‐dependent stratification, consistent with previous findings (log‐rank *p* < 0.001) [45]. Cox regression further confirmed this, with the highest risk in patients with > 10 years of T2DM (adjusted HR = 4.37), similar to other reports but higher than earlier estimates (e.g., HR = 2.8), likely due to more robust adjustment for confounders such as HbA1c and LVEF [46, 47]. The 5–10‐year group showed a nonsignificant risk increase in unadjusted analysis but reached significance after adjustment, underscoring the importance of controlling for key covariates, a limitation in earlier studies [48]. Although our follow‐up was shorter, our findings are consistent with larger, long‐term studies [40].

This study has several limitations. The relatively small sample size may have limited statistical precision, particularly in subgroups with extended durations of T2DM. There is a possibility that the follow‐up duration was inadequate to capture long‐term effects, such as the emergence of microvascular complications or late stent thrombosis. In particular, the relatively small number of events in the > 10‐year diabetes duration group (*n* = 23) resulted in wide CIs, indicating some degree of imprecision in the risk estimates. Therefore, these findings should be interpreted with caution. Furthermore, the single‐center design and predominance of male participants (approximately 71%) may limit the generalizability of the results to other populations, particularly women and broader South Asian settings. Moreover, only baseline HbA1c levels were considered, and the absence of longitudinal glycemic control data during follow‐up may have resulted in residual confounding. A formal post hoc power calculation was not performed; however, the observed statistically significant associations suggest that the study had adequate power to detect clinically meaningful differences.

Future research should incorporate larger, multicenter prospective studies to enhance generalizability and validate findings. Such studies would also help improve the precision of effect estimates and confirm the observed associations. Extended follow‐up periods would shed light on how T2DM duration influences MACE and other complications following PCI in the long term. Investigating tailored treatment strategies based on glycemic control, genetic factors, and the duration of T2DM may improve patient outcomes. The relationship between microvascular disease, T2DM duration, and MACE should be further examined. Ultimately, addressing these gaps will improve therapeutic outcomes and reduce the incidence of CVD in this high‐risk population.

In conclusion, a clear linear, graded association was observed between T2DM duration and the risk of MACE following PCI, with event rates increasing from 3.7% in patients with diabetes < 5 years to 13.9% in those with > 10 years. This pattern suggests a progressive increase in cardiovascular risk with longer duration of diabetes. These findings reinforce the prognostic significance of diabetes chronicity and highlight the importance of incorporating duration of diabetes into risk stratification and long‐term management strategies for patients with T2DM.

## Author Contributions

Conceptualization, methodology, software, formal analysis, investigation, data curation, and writing original draft preparation, A.T.; validation, Z.B., A.H.K., M.A., and M.L.; writing, review, and editing, B.A.T., K.H., and M.A.; visualization, supervision, and project administration, Z.B.

## Funding

No funding was received for this manuscript.

## Disclosure

All authors have read and agreed to the published version of the manuscript.

## Ethics Statement

This study was conducted in accordance with the Declaration of Helsinki. Ethical approval was obtained from the Institutional Research Ethics Committee (IREC) of the Faculty of Pharmacy, University of Lahore (Ref. No: IREC‐2023‐5H/18‐09‐2023) and the Punjab Institute of Cardiology, Lahore, Pakistan (Ref. No: PH‐Research‐186/20‐09‐2023).

## Conflicts of Interest

The authors declare no conflicts of interest.

## Data Availability

The datasets used and/or analyzed during the current study are available from the corresponding author upon reasonable request.
